# A Manual of Elementary Zoology

**Published:** 1913-03

**Authors:** 


**A Manual of Elementary Zoology.** By L. A. Borradaile, M.A. Pp. ix, 470. London: Henry Frowde and Hodder & Stoughton. 1912. 10s. 6d. net.

This book is written apparently not with the intention of covering any known syllabus, but with the idea of giving the student an introduction to those animal forms commonly used in the laboratory. It seems a pity that so much space should be given up to a detailed description of the frog, when we already have such excellent books as the new and revised edition of Marshall's *Frog*.

Several features seem to us to be worthy of special note, and we must congratulate the author on his departure from many of the text-figures that have done yeoman service in previous books. Some of the diagrams, however, could be improved upon, *e.g.* the third figure on page 183, and also the figure which illustrates the scales of the dog-fish.

It is a matter for regret that such a small space has been given to a consideration of the protozoa and of the insecta, when we consider their importance to the medical student. It must be remembered that the medical student will never have an opportunity of getting a thorough grasp of such forms at any later period in their course, which is already tremendously overcrowded.

A few features in the book might with advantage be carefully considered when a new edition is being prepared. For example, would it not be better to return to the more generally accepted spelling of the name paramœcium? Again, we feel inclined to believe that the diagram representing the changes that occur in the nucleus of paramœcium during conjugation is not quite accurate, and that showing the venous system of the dog-fish is also doubtful. We have also grave doubts as to the real nature of the artery spoken of as the hyoid artery in the dog-fish, and of that foramen which is called the obturator foramen in the rabbit.

We would suggest to the author that in a subsequent edition part of the space at present allocated to the frog should be given up to a brief consideration of some of the more general problems of zoology. In spite of these few criticisms, we must heartily congratulate the author on the production of the book.

